# Supplementary material for: Is It Possible to Create Antimicrobial Peptides Based on the Amyloidogenic Sequence of Ribosomal S1 Protein of P. aeruginosa?
Source: Int J Mol Sci. 2021 Sep 10;22(18):9776. doi: 10.3390/ijms22189776 (PMC8469417; doi:10.3390/ijms22189776)
Supplement: Supplementary file 1 [file ijms-22-09776-s001.zip › ijms-1363770-supplementary.pdf]

**Table S1.** Results of determination of the antibacterial properties of peptide R12-Sar (RKKRRQRRRGG-Sar) with *P. aeruginosa* (strain ATCC 9027) cells on agar.

| | 3.75 μM | 37.5 μM | 375 μM | 3750 μM | 20% (volume/volume) DMSO | Control |
|---|---|---|---|---|---|---|
| *P. aeruginosa*, strain ATCC 9027 | Non-AMP | Non-AMP | Non-AMP | Non-AMP | – | – |

**Table S2.** Results of determination of the antibacterial properties of peptide R12-Sar (RKKRRQRRRGG-Sar) with *P. aeruginosa* (strain PA103) cells on agar

| | 6.56 μM | 65.6 μM | 656 μM | 6560 μM | 100% (volume/volume) DMSO | 20% (volume/volume) DMSO |
|---|---|---|---|---|---|---|
| *P. aeruginosa*, strain PA103 | Non-AMP | Non-AMP | Non-AMP | Non-AMP | – | – |

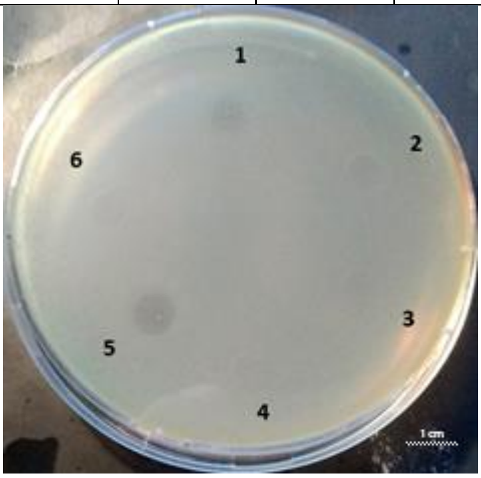


**Figure S1**. Inhibition zone assay after 24 h of co-incubation peptide R12-Sar (RKKRRQRRRGG-Sar) with *P. aeruginosa* (strain PA103) cells on agar. The test for the antibacterial effect of peptide R12-Sar was carried out according to the following scheme: 1 – peptide 6560 μM, 2 – peptide 656 μM, 3 – peptide 65.6 μM, 4 – peptide 6.56 μM, 5 – DMSO 100% (volume/volume), 6 – DMSO 20% (volume/volume). The scale bar is 1 cm.
